# Feeding Practices and Dietary Diversity in the First Year of Life: PreventADALL, a Scandinavian Randomized Controlled Trial and Birth Cohort Study

**DOI:** 10.1016/j.tjnut.2023.06.015

**Published:** 2023-06-17

**Authors:** Carina Madelen Saunders, Eva Maria Rehbinder, Karin C. Lødrup Carlsen, Christine Monceyron Jonassen, Marissa LeBlanc, Björn Nordlund, Håvard Ove Skjerven, Cilla Söderhäll, Riyas Vettukattil, Monica Hauger Carlsen

**Affiliations:** 1 University of Oslo, Faculty of Medicine, Institute of Clinical Medicine, Oslo, Norway; 2 Division of Paediatric and Adolescent Medicine, Oslo University Hospital, Oslo, Norway; 3 Department of Dermatology, Oslo University Hospital, Oslo, Norway; 4 Genetic Unit, Centre for Laboratory Medicine, Østfold Hospital Trust, Kalnes, Norway; 5 Faculty of Chemistry, Biotechnology and Food Science, Norwegian University of LifeSciences, Ås, Norway; 6 Oslo University Hospital and University of Oslo, Oslo, Norway, Oslo Centre for Biostatistics and Epidemiology; 7 Astrid Lindgren Children’s Hospital, Karolinska University Hospital, Stockholm, Sweden; 8 Department of Women’s and Children’s Health, Karolinska Institutet, Stockholm, Sweden; 9 Department of Nutrition, Institute of Basic Medical Sciences, University of Oslo, Oslo, Norway

**Keywords:** breastfeeding, complementary food, solid food, food diversity, diet diversity, early food introduction, infant diet, infant feeding, infant feeding practices, PreventADALL

## Abstract

**Background:**

Breastmik is considered the optimal source of nutrition in early infancy. However, recommendations and practices for when and how complementary food should be introduced in the first year of life vary worldwide. Early introduction of allergenic foods may prevent food allergies, but if early food introduction influences infant feeding practices is less known.

**Objectives:**

We sought to assess infant feeding practices in the first year of life and to determine if early interventional food introduction influences breastfeeding and dietary diversity.

**Methods:**

Dietary intake was assessed in infants from the population-based clinical trial Preventing Atopic Dermatitis and ALLergies (PreventADALL) in children study. A total of 2397 infants were cluster-randomized at birth into 4 different groups: *1*) control, *2*) skin intervention, *3*) introduction to 4 allergenic foods between 3 and 4 mo of age: peanut, cow milk, wheat, and egg, as small tastings until 6 mo, and *4*) combined skin and food interventions. Dietary data were available from at least one of the 3-, 6-, 9-, and 12-mo questionnaires in 2059 infants. In the present analysis, groups 1 and 2 constitute the No Food Intervention group, whereas groups 3 and 4 constitute the Food Intervention group.

We used the log-rank test and Cox regression to assess the impact of food intervention on age of breastfeeding cessation. Mixed effects logistic regression was used to compare dietary diversity, defined as the number of food categories consumed, between intervention groups.

**Results:**

At 3, 6, 9, and 12 mo, 95%, 88%, 67%, and 51% were breastfed, respectively, and breastfeeding duration was not affected by the food intervention. In the No Food Intervention group, mean age of complementary food introduction was 18.3 wk (confidence interval [CI]: 18.1, 18.5). In the Food Intervention group, the dietary diversity score was 1.39 units (CI: 1.16, 1.62) higher at 9 mo (*P* < 0.001) and 0.7 units (CI: 0.5, 0.9) higher at 12 mo (*P* < 0.001) compared to the No Food Intervention group.

**Conclusions:**

Early food intervention did not affect breastfeeding rates and increased dietary diversity at 9 and 12 mo.

## Introduction

The first year of life is a critical period to establish feeding practices, taste preferences, and long-term dietary habits. Breastmilk is considered the optimal source of nutrition for infants [1,2]. Exclusive breastfeeding for ≥4 mo is recommended by European authorities [3,4] and for 6 mo by the American Academy of Pediatrics and WHO [5,6]. In Norway, exclusive breastfeeding is recommended for the first 6 mo of life, with an optional introduction of complementary foods (foods other than breastmilk or formula) from 4 mo, if breastmilk is insufficient [7,8].

Compared to other countries, Norway has high rates of breastfeeding initiation at birth (98%), with 80% of infants still breastfeeding at 6 mo, and many continuing to breastfeed throughout the first year of life [9]. Most infants are introduced to complementary foods by 6 mo of age [[10], [11], 12]]. Breastfeeding rates in Sweden are lower than that in Norway but have been increasing in recent years, with 63% breastfeeding at 6 mo and 28% at 12 mo [13,14].

Throughout the years, recommendations regarding the introduction of complementary foods have changed and continue to be a topic of debate [15,16]. These challenges are reflected in great variations of infant feeding practices between industrialized countries [17]. Several randomized controlled trials (RCTs), including the Preventing Atopic Dermatitis and ALLergies (PreventADALL) trial, found evidence that earlier introduction of specific allergenic foods may prevent allergic sensitization and food allergies [[18], [19], [20], [21]]. In response, a number of guidelines have been adapted [[22], [23], [24]].

A diverse diet and timely introduction of complementary foods may also ensure an adequate supply of nutrients and influence both short- and long-term health outcomes [[25], [26], [27]]. As long as complementary foods are given in an age-appropriate texture and are nutritionally adequate, there is currently no convincing evidence that early food introduction has adverse health effects [3]. Dietary diversity is defined as the number of different foods or food groups consumed over a given period [28]. Dietary diversity is linked to a higher nutrient intake and increased exposure to different food antigens [29]. A diverse diet could decrease the risk of developing food allergies [26] and may also have positive health effects by exposing the gut microbiota to various foods early on and thus increase microbial diversity [[30], [31], [32]].

Given the conflicting advice and cultural differences in infant feeding practices, more research into the timing of complementary food introduction is warranted. Therefore, the primary aim of this substudy, from the Scandinavian RCT and birth cohort PreventADALL, was to assess infant feeding practices, including the timing of complementary food introduction. The secondary aim was to determine if early food intervention (early exposure to small tastes of allergenic foods) impacted breastfeeding rates and dietary diversity in the first year of life.

## Methods

### Study design and study population

In the present study, we analyzed dietary data from infants in the PreventADALL RCT, a multicenter, prospective, general, population-based mother–child birth cohort, aiming at primary prevention of allergic disease. Study design, recruitment, inclusion criteria, and baseline characteristics are described in detail elsewhere [33]. Briefly, 2397 mother–child pairs were recruited from the general population of pregnant women around 18 wk of gestational age at Oslo University Hospital, Østfold Hospital Trust (Norway), and Karolinska University Hospital (Stockholm, Sweden) between December 2014 and October 2016. The children were included at birth, provided there was no severe illness and gestational age was ≥35.0 wks. Three children later withdrew from the study. Maternal health and sociodemographic and lifestyle factors were obtained through electronic questionnaires administered during pregnancy, and detailed electronic questionnaires, including infant dietary data, were obtained at 3 (77% response rate), 6 (71% response rate), 9 (68% response rate), and 12 (69% response rate) mo of age. We included all 2059 infants with at least one completed diet questionnaire.

Newborns were randomly assigned to 4 intervention groups: no intervention (control group), skin intervention, food intervention, or the combined interventions [34]. The control group was instructed to follow regular advice and national guidelines regarding weaning and skin care. The skin intervention consisted of a 5- to 10-minute emulsified oil bath and face cream (Ceridal) for ≥4 d/wk, from 2 wk through 8 mo of age. The food intervention consisted of small tastes of 4 different commonly allergenic foods between 3 and 4 mo of age. Peanut butter was given for the first time at the 3-mo follow-up visit. Cow milk was introduced 1 wk later, followed by wheat, and scrambled eggs in the fourth week of introduction. Parents were instructed to give each of these foods as a tiny taste, either from their finger or a teaspoon, ≥4 d/wk and until 6 mo without any dose restriction. The purpose of the early food intervention was to expose infants to small amounts of allergenic foods over time and was not intended to replace breastmilk.

The PreventADALL study was approved by the Regional Committee for Medical and Health Research Ethics in South-Eastern Norway (2014/518) and in Stockholm, Sweden (2014/2242-31/4). The study was registered at clinicaltrials.gov (NCT02449850). Informed consent was given upon enrollment and inclusion of the infant at birth.

Dietary data were obtained through electronic weekly diaries (2–26 wk of age) and questionnaires (at 3, 6, 9, and 12 mo of age). Breastfeeding was assessed at 3, 6, 9, and 12 mo of age by parents reporting for the last 3 mo if the infant had received breastmilk and the time of breastfeeding cessation. If the infant was not exclusively breastfed, the amount of breastmilk consumed was categorized as most of the diet, the same amount as other food, or as a small part of the diet. At 6 mo, parents reported the amount of breastmilk consumed as no breastmilk, a small part of the diet, approximately half of the diet, or most of the diet. Consumption of porridge, how often it was given, at what age it was first introduced, and what it was made of (rice, millet, oat, corn, wheat, whole meal, spelt, Sinlac, and other types) was reported at 3 and 6 mo. Questions about dairy intake at 6, 9, and 12 mo included the time of first introduction and what kind of dairy products were given to the infant. At 6, 9, and 12 mo, parents were asked to indicate how often the infant consumed several solid foods. The categories included: bread/cookies/waffles/cakes and other baked goods, fruit or berries, root vegetables (such as potato, turnip, carrot, and parsnip), other vegetables, peanut (as a spread or incorporated in other foods), pure egg (eg, fried, cooked, scrambled, and eggnog), egg in other foods (eg, gratin, waffles, baked goods, paste, or similar), fatty fish (salmon, trout, mackerel, pike, halibut, and eel), other fish, shellfish, poultry meat, and other meat. We also asked about how much of the infant food was home cooked compared with commercially prepared (industrially processed), as well as the content of organic food in the infant’s diet.

All questions about diet were mandatory, assuring a complete set of values in all questionnaires. Please see the Supplementary data section and Supplementary Figure 1 for more details on the infant diet questions.

### Definitions

**FIGURE 1:**
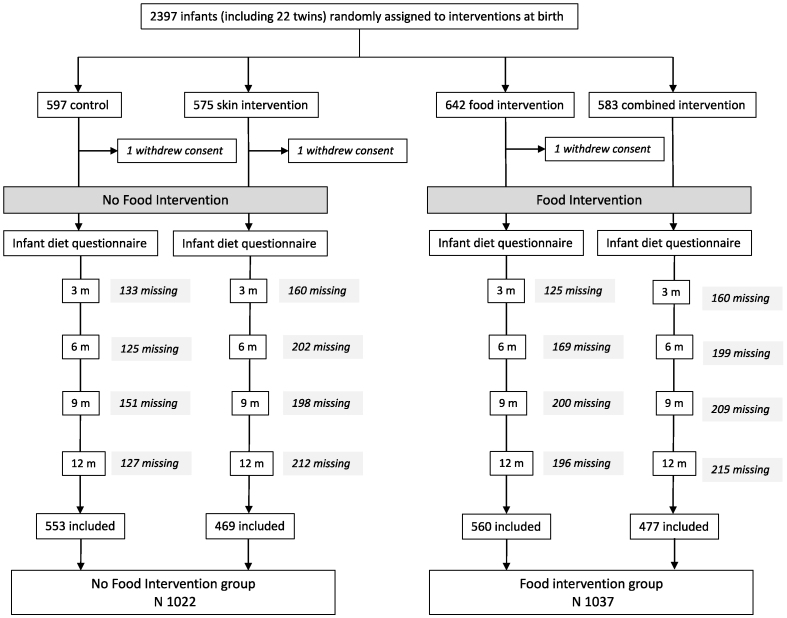
Overview of the PreventADALL study population, including withdrawals and randomization groups. We included all 2059 infants with at least one completed dietary questionnaire. In the present study, the Food Intervention group includes infants from both the food intervention and combined intervention group, whereas the No Food Intervention group includes infants from the control and skin intervention groups.

In the present analysis, we included all infants who were randomly assigned to the food intervention (early exposure to small amounts of 4 allergenic foods [peanut, cow milk, wheat, and egg] as part of the study intervention described earlier [33]) into the Food Intervention (FI) group, whereas the No Food Intervention (NFI) group consisted of all study participants who were randomly assigned to no food intervention (control group and the skin intervention groups). For an overview of the study population, see Figure 1.

Complementary food introduction was defined as the age at which any food or drink other than breastmilk, formula, and water was given for the first time to the infant.

#### Outcomes

Primary outcome: Breastfeeding duration was defined as the number of months an infant was breastfed. Breastfeeding rates included exclusively breastfed infants and partially breastfed infants (receiving breastmilk and other sources of nutrition).

Secondary outcome: The dietary diversity score is defined as the number of different food groups given to the infant during the last 3 months at 6, 9, and 12 mo of age. The calculated score was based on the following food groups: bread and other baked goods, milk, fermented dairy products, cheese, other dairy products, fruits and berries, root vegetables, other vegetables, peanuts, other nuts, egg (pure and egg in other foods), fatty fish, other fish, shellfish, poultry, and other meat. Summing the number of foods introduced at 9 and 12 mo, the maximum score was 16. At 6 months, the maximum score was 10 because intervention foods, including all dairy products, peanuts, and egg, were excluded from the dietary diversity score. Infant porridge was not included in the score because we did not include questions on infant porridge in the 9- and 12-mo questionnaires. The dietary diversity score did not consider frequency or portion sizes.

### Statistical analysis

Categorical variables are presented as numbers and percentages. Continuous variables, including frequency and intake of various foods, are presented as means, SD, and minimum (min) – maximum (max). To analyze breastfeeding rates in the first year of life, we used Cox regression. Kaplan–Meier curves were estimated for the FI and NFI groups, and differences in cessation rates between intervention groups were compared using the log-rank test. The proportional hazards assumption was tested and met. These analyses were performed using IBM SPSS statistics version 25.

We used R version 3.6.0 for dietary diversity analysis, including complete case (ie, answered the dietary questionnaire at a given time point) and modified intention to treat analysis (ie, all randomly assigned). For dietary diversity assessment at each time point between the intervention groups, we analyzed dichotomous end points using mixed effects logistic regression with the interventions and their interaction as fixed effects and randomization period and residential postal code as random effects.

For the intention to treat analysis, missing primary outcome data were imputed using multiple imputations by chained equations. The number of multiple imputations was 15, and the scalar giving the number of iterations was 20. Complete case analysis was conducted as a sensitivity analysis.

## Results

The background characteristics of the 1022 infants in the FI group and 1037 infants in the NFI group (N1037) were similar, as shown in Table 1. We also assessed the background characteristics among our study population of 2059 infants and their parents and the remaining 338 study participants in the PreventADALL cohort, as seen in Supplementary Table 1. In our study population, 440 (21%) were from Sweden and 1619 (79%) from Norway.

**TABLE 1:** Baseline characteristics of study population^1^

		NFI group (*n* = 1022)	FI group (*n* = 1037)
Age of mother, y		32.4 (4.1, 21–48)	32.6 (4.0, 20–44)
Age of father, y		34.8 (5.4, 23–72)	34.8 (5.5, 21–72)
Male sex, infants		516 (51)	562 (54)
Birth weight, g		3582 (489, 1794–5632)	3573 (463, 2005–4900)
Birth length, cm		50.5 (2.2, 33–61)	50,5 (2.0, 44–56)
Vaginal delivery		861 (84)	865 (83)
Caesarian section		161 (16)	172 (17)
Marital status^2^	Married	385 (41)	400 (42)
	Cohabitants	530 (56)	541 (56)
	Single	19 (2)	15 (2)
	Divorced/separated	0 (0)	0 (0)
	Other	5 (<1)	9 (1)
Maternal education level^3^	Primary school (9/10 y)	6 (<1)	5 (<1)
	High school only	86 (9)	96 (10)
	Higher education <4 y	288 (31)	302 (31)
	Higher education ≥4 y	520 (56)	535 (56)
	PhD	32 (3)	24 (3)
	Other education	2 (<1)	0 (0)
Paternal education level^4^	Primary school (9/10 y)	10 (1)	12 (1)
	High school only	163 (18)	169 (18)
	Higher education <4 y	261 (29)	283 (31)
	Higher education ≥4 y	426 (47)	423 (46)
	PhD	32 (4)	31 (3)
	Other education	3 (<1)	1 (<1)
Maternal work	Full time	808 (79)	821 (79)
	Part-time	85 (8)	83 (8)
	Student	55 (5)	60 (6)
	Housewife/homemaker	7 (<1)	9 (<1)
	Jobseeker/unemployed	9 (<1)	11 (1)
	Disabled	3 (<1)	7 (<1)
	Other	13 (1)	18 (2)
Household income (NOK)^5^	Below 300,000	9 (1)	8 (1)
	300,000–600,000	117 (13)	112 (12)
	600,000–1,000,000	383 (41)	396 (41)
	1,000,000–1,400,000	299 (32)	319 (33)
	>1,400,000	119 (13)	112 (12)
	Did not want to answer	12 (1)	18 (2)
Tobacco use (ever)	Smoking	197 (22)	213 (21)
	Snus	205 (22)	218 (23)

Abbreviations: FI, Food Intervention; NFI, No Food Intervention.

1 Values are means (SD, min-max) or *n* (%) unless otherwise stated.

2 Information available from *n* = 939 (NFI)/*n* = 965 (FI)

3 *n* = 934 (NFI)/*n* = 962 (FI),

4 *n* = 906 (NFI)/*n* = 924 (FI)

5 *n* = 939/*n* = 965

### Breastfeeding and complementary feeding

In the Swedish population, breastfeeding rates at 3 mo and 6 mo were 92% and 80% compared with 95% and 88% in the Norwegian part of the study population, respectively. At 12 mo, 22% breastfed their children in Sweden and 51% in Norway.

In the NFI group, the mean age of complementary food introduction was 18.3 wk (CI: 18.1, 18.5 wk), whereas infants randomly assigned to food intervention were introduced complementary foods from 12 wk of age. Porridge was the most common first food, other than the interventional foods (peanut, cow milk, wheat, and egg). In the total study population, porridge was introduced in 9.6 % of infants by 3 mo of age, 45.7% at 4 mo, and 25.6% at 5 mo. By 6 mo of age, 81.1% infants had been given porridge. The most frequent porridges to be introduced before 6 mo were oat (62.2%), wheat (40.5%), and corn (34.1%). The following porridge types were given less frequently: millet (18.3%), whole meal (13.3%), spelt (4.1%), Sinlac (4.2%), and other types (5.1%).

The cumulative proportion of infants introduced to other complementary foods is listed in Table 2. The most common complementary foods given at 6 mo of age were fruit and berries, root vegetables, and other vegetables. Most infants consumed dairy (in any form) by 12 mo of age. The number of infants consuming fish, meat, and poultry increased from 6 to 9 mo and were consumed by most infants by 12 mo of age.

**TABLE 2:** Proportion of infants who received various complementary foods at 6, 9, and 12 mo in the No Food Intervention and Food Intervention groups^1^

	6 mo (%)		9 mo (%)		12 mo (%)	
	NFI	FI	NFI	FI	NFI	FI
Cow milk	11	64 ∗	30	64 ∗	58	74 ∗
Lactose free milk	<1	3 ∗	4	7 ∗	7	8
Unpasteurized cow milk	<1	<1	<1	2 ∗	1	2
Pro/prebiotic dairy	2	7 ∗	6	13 ∗	20	23 ∗
Yogurt	12	26 ∗	41	60 ∗	80	85 ∗
Cheese	6	8 ∗	50	62 ∗	86	88
Other dairy products	8	14 ∗	36	48 ∗	58	63 ∗
Bread and other baked goods	21	28	88	90 ∗	96	97
Fruit and berries	92	93	99	99	99	99
Root vegetables	89	85	99	99	99	99
Vegetables	69	66	97	97	98	98
Peanuts	12	85 ∗	22	72 ∗	34	68 ∗
Other nuts	4	7 ∗	15	21 ∗	24	35 ∗
Egg foods	11	22 ∗	63	76 ∗	87	90 ∗
Egg pure	21	82 ∗	64	82 ∗	81	89 ∗
Fatty fish	19	17	86	86	93	93
Fish other	12	12	70	70	84	84
Shellfish	1	3	12	13	27	29
Poultry	18	15 ∗	81	81	88	88
Meat other	22	20	90	89	94	94
Home-cooked vs commercially prepared						
Only home-cooked	9	9	4	4	4	3
>Home-cooked^2^	21	22	24	24	34	36
Equal amounts	23	22	25	24	31	28
> Commercially prepared	34	36	43	42	29	31
Only commercially prepared	12	10	4	6	2	2
Amount of organic food						
Very little/ none	15	16	8	11	11	14 ∗
Small amount	21	22	29	28	38	37
Half	23	23	29	28	28	28
Majority	32	29	26	25	18	16
Don’t know	9	9	5	7	6	5

Abbreviations: FI, Food Intervention; NFI, No Food Intervention.

1 Numbers are presented in %.

2 > Home cooked refers to the infant diet consisting of more home-cooked foods than commercially prepared infant foods, and vice versa for > Commercially prepared.

∗ Indicates a significance *P* < 0.05 when comparing infants in the Food Intervention (FI) group with infants in the No Food intervention (NFI) group at each time point. We used chi-square test to assess differences of complementary food intake between groups.

At 6 mo, 45% of infants were reported to consume mostly or only commercially prepared infant foods, whereas 9% consumed only home-cooked foods. By 12 mo, the proportion of home-cooked foods in the infant’s diet increased. The proportion of infants mainly receiving organic food declined from 6 to 12 mo (Table 2).

### Impact of early food intervention on breastfeeding rates and dietary diversity

**FIGURE 2:**
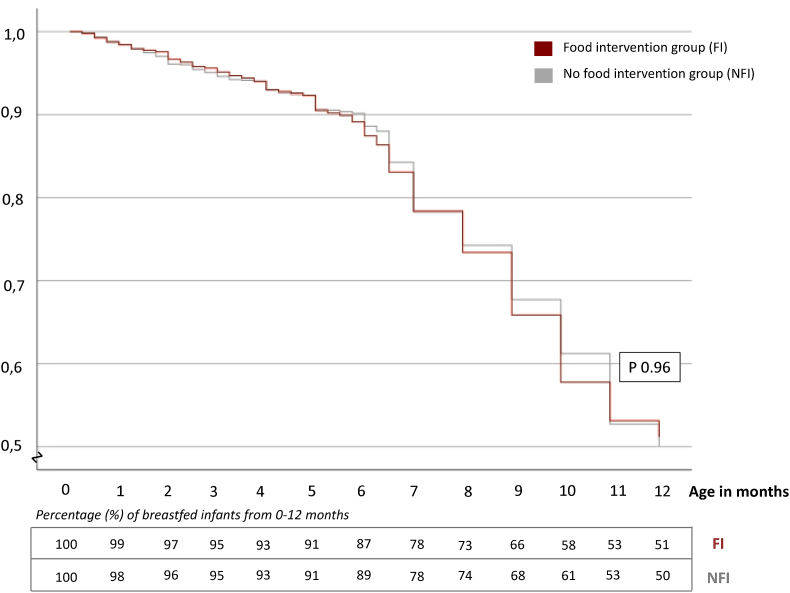
Kaplan–Meier curve showing no significant difference in breastfeeding rates (%) between the Food Intervention and No Food Intervention groups from 0 to 12 mo in 2059 infants. The table shows the percentage of breastfed infants each month in both groups from 0 to 12 mo.

Breastfeeding rates from birth until 12 mo did not differ significantly between the FI (early introduction of peanut, milk, wheat, and egg) and the NFI group (*P* = 0.96), as shown in Figure 2.

Significantly fewer infants in the NFI group consumed dairy products, peanuts, other nuts, egg foods, and pure egg at 6 mo compared with infants in the FI group (*P* < 0.001). At 9 and 12 mo, the percentage of infants consuming these foods remained lower (*P* < 0.001) in the NFI group compared with the FI group (Table 2). Intakes of other complementary foods were not influenced by the intervention, except fewer infants in the FI group consumed poultry at 6 mo (*P* = 0.032) and more infants consumed bread and other baked goods at 9 mo (*P* = 0.043). Intake of commercially prepared infant foods compared with home-cooked foods was similar in both groups, as was the intake of organic foods, except more infants in the FI group consumed little or no organic food at 12 mo.

**FIGURE 3:**
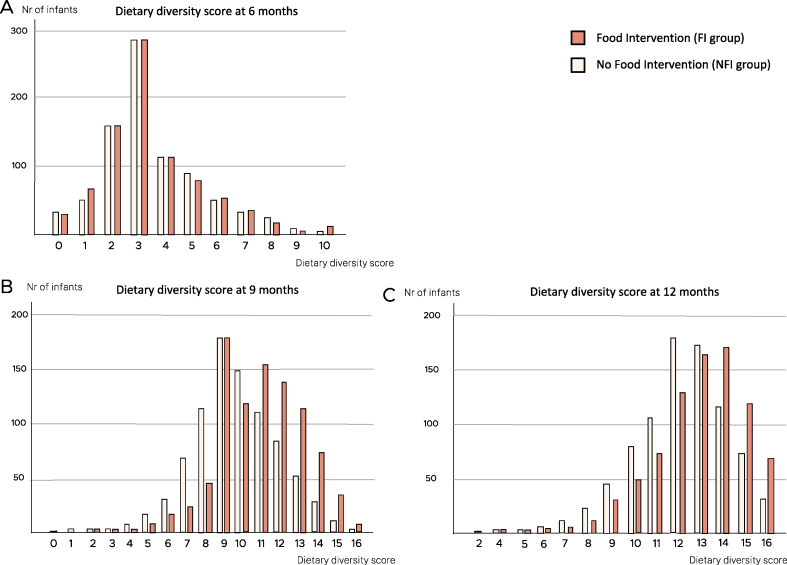
Dietary diversity score in infants categorized by Food Intervention compared with No Food Intervention groups at 6, 9, and 12 mo of age. The x-axis represents the total number of food items introduced. The y-axis presents the number of infants. (A) Dietary diversity score at 6 mo (*n* = 1700). (B) Dietary diversity score at 9 mo (*n* = 1637). (C) Dietary diversity score at 12 mo (*n* = 1645).

Excluding interventional foods, the mean dietary diversity score (number of food categories consumed) was similar in the FI compared with the NFI group at 6 mo (3.4 [SD 1.9] compared with 3.5 [SD 1.9], respectively). However, the dietary diversity score was significantly higher in the FI group compared with the NFI group at 9 and 12 mo of age (*P* < 0.001), as shown in Table 3 and Figure 3. The dietary diversity score, including interventional foods, was 11.1 (SD 2.3) in the FI group compared with 9.7 in the NFI group (SD 2.4) at 9 mo and 12.9 (SD 1.2) compared with 12.1 (SD 2.1) at 12 mo.

**TABLE 3:** Effect of early interventional food introduction on dietary diversity score

	6 mo			9 mo			12 mo		
	Effect estimate	CI	*P* value	Effect estimate	CI	*P* value	Effect estimate	CI	*P* value
Linear regression, multiple imputation	0.01	−0.18, 0.20	0.95	1.39	1.16, 1.62	<0.001	0.70	0.50, 0.90	<0.001
Linear regression, complete case^1^	−0.28	−0.71, 0.15	0.77	1.41	1.18, 1.64	<0.001	0.78	0.58, 0.98	<0.001

1 no imputation

## Discussion

In a general population of >2000 infants, >85% of infants were breastfed throughout the first 6 mo of life, and half of the population was still breastfed at 12 mo. The mean age of complementary food introduction in the NFI group was 4.5 mo. Most infants were introduced to complementary foods, mainly fruits, vegetables, and porridge, between 4 and 6 mo. The food intervention did not affect breastfeeding rates, whereas the dietary diversity score at 9 and 12 mo was significantly higher in infants subjected to the early food intervention compared with the infants who were not.

Breastfeeding rates in our study are consistent with recent findings in Spedkost 3, a Norwegian nationwide survey from 2019, including 2182 infants [9,35], and higher than in another study from 2013, including 2500 infants, where 35% were breastfed at 12 mo [8,9]. Our results indicate that Norwegian breastfeeding rates have experienced an incline in the past decade, with a notable increase in breastfeeding from 3 to 9 mo [12]. In our study, breastfeeding rates were considerably lower in the Swedish study population, consistent with surveys based on medical records of children born in Sweden in 2017, where 44% were breastfed at 9 mo and 27% at 12 mo [36]. The shorter breastfeeding duration in Sweden might be a result of differences in parental leave policies [37].

A study from 2019 [38] of 11 European countries found that 56%–98% of infants were breastfed directly after birth and 38%–71% at 6 mo, with 10%–38% being exclusively breastfed at 6 mo. At 6 mo of age, Norway (71%), Sweden (61%), and Germany (57%) reported the highest rates of any breastfeeding.

Most infants in the NFI group were introduced to complementary foods between 4 and 6 mo of age, in line with the European Society for for Paediatric Gastroenterology, Hepatology and Nutrition (ESPGHAN) guidelines [4] and the Spedkost 3 study in Norway, with 62% of infants introduced to complementary foods at 4 mo and 98% by 6 mo [9]. The rate of complementary food introduction before 4 mo has decreased from 21% in 1998 to 11% in 2006, 7% in 2013, and 6% in 2019 [12,39]. Complementary food is introduced earlier in other countries, with recent rates of complementary feeding before 4 mo being 21% in 2157 Dutch infants, 32% in the United States [40], 43% in the United Kingdom, and 40% in New Zealand [41,42]. The intake of commercially prepared infant foods and organic food at 6 and 9 mo in our population is comparable to the findings in Spedkost 3 [9].

Breastfeeding rates in our study were not affected by the food intervention, similar to 2 other large early allergenic food introduction RCTs, the Enquiring About Tolerance study in a general population [20] and the Learning Early About Peanut Allergy trial in children at high risk of peanut allergy [43]. In contrast, a recent Swedish study of 1200 infants with dietary information reported at 1 y of age showed that earlier exposure to tiny tastings of complementary foods was associated with shorter breastfeeding length [44]. Another study, including 856 children from the Protection Against Allergy Study in Rural Environments/EFRAIM study, found no association between breastfeeding duration and diet diversity [45].

The finding that early introduction of complementary foods was associated with significantly increased dietary diversity at 9 and 12 mo of age is novel, to the best of our knowledge. Dietary diversity in infancy has gained significant attention because of its role in modulating allergic disease outcomes. Given the complexities and challenges of dietary diversity research, the European Academy of Allergy and Clinical Immunology Task Force recently provided an expert consensus regarding multiple aspects of diet diversity research and highlighted the need for more clinical trials with agreed definitions [26].

Major strengths of the present study include the prospective design and robust sample size from a general population and the systematic collection of detailed questionnaires, which limits the likelihood of recall bias and ensures high accuracy of timing of solid food introduction and milk-feeding practices [46]. Response bias may be an issue with self-reporting; however, we found similar results in our main analysis using imputed data for missing dietary diversity and in the sensitivity analysis, thereby strengthening our findings. Comparisons with previous national dietary surveys need to be made with caution because of differences in study cohort selection and questionnaires. Dietary diversity is a challenging area of research because single nutrients or foods are not eaten in isolation but are part of a complex interplay of factors. Considerations should also be given to diversity in diets being strongly impacted by ethnic traditions, leading to unmeasurable characteristics that could influence the outcome and interpretation of the results. The dietary diversity score could have been influenced by certain limitations in our questionnaires that did not assess certain food groups, such as legumes, and the complementary foods given were not quantified. Our study population consisted of a larger proportion of infants with a first-degree relative with allergic disease, higher maternal educational level, and higher income than the general population of Norway and Sweden, which might have influenced feeding practices.

The Norwegian Directorate of Health recommends exclusive breastfeeding for 6 months, in line with the WHO [5,8]. These recommendations are particularly important in low-income countries where childhood malnutrition is prevalent. European infants are less likely to experience deficiency of nutrients in the complementary feeding period [4]. High-income countries face different challenges, including an increase in allergic diseases, a loss of gut microbial diversity, and impaired immune development. Potential mechanisms by which a higher dietary diversity could protect against allergic and other noncommunicable diseases is through modulation of the infant’s gut microbiota, increased nutrient intake, and exposure to different food antigens [26,30,47].

An infant’s readiness to start complementary feeding will depend on the individual’s development [3,4]. Evidence suggests that renal and gastrointestinal functions are sufficiently mature at 4 mo to metabolize complementary foods [4]. Because neuromotor development and apparent interest in nonmilk foods differ among infants, the focus should be on determining an appropriate age range for complementary food introduction [3].

As breastfeeding is the best for the infant, early introduction of solid foods should not come at the expense of breastfeeding. The results from our study suggest that early complementary introduction is beneficial for increased dietary diversity with no negative impact on breastfeeding rates. Further studies are needed to address the potential impact on disease prevention, as well as potential underlying mechanisms.

In summary, in this general population-based mother–child birth cohort, most infants were breastfed throughout the first 6 mo of life, and half were still breastfed at 12 mo. The mean age of complementary food introduction was 4.5 mo, and most infants received complementary foods by 6 mo. Our data provide novel information that introducing small amounts of complementary foods from 3 mo of age increased dietary diversity but not at the expense of breastfeeding rates or breastfeeding duration.

## Author contributions

The authors’ responsibilities were as follows – CMS, EMR, KLC, HOS, MHC: designed research; CMS, EMR, KLC, CMJ, MLB, BJ, HOS, CS, RV, MHC: conducted research; CMS, MLB, RV: analyzed data or performed statistical analysis; CMS: wrote paper, CMS, EMR, MHG: had primary responsibility for final content; and all authors: read and approved the final manuscript.

## Conflict of interest

CMS reports personal fees from Libero outside the submitted work. ML reports personal fees from MSD outside the submitted work. EMH reports personal fees from Sanofi-Genzyme, Novartis, Leo-Pharma, Perrigo, and The Norwegian Asthma and Allergy Association outside the submitted work. All other authors report no conflicts of interest.

## Funding

The PreventADALL study was supported by the following sources: The Regional Health Board South East, The Norwegian Research Council, Oslo University Hospital, The University of Oslo, Health and Rehabilitation Norway, The Foundation for Healthcare and Allergy Research in Sweden – Vårdalstiftelsen, The Swedish Asthma- and Allergy Association’s Research Foundation, The Swedish Research Council – the Initiative for Clinical Therapy Research, The Swedish Heart-Lung Foundation, SFO-V Karolinska Institutet, Østfold Hospital Trust, by unrestricted grants from the Norwegian Association of Asthma and Allergy, The Kloster foundation, Thermo-Fisher, Uppsala, Sweden (through supplying allergen reagents) and Fürst Medical Laboratory, Oslo, Norway (through performing IgE analyses), Norwegian Society of Dermatology and Venerology, Arne Ingel’s legat, Region Stockholm (ALF-project and individual grants), Forte, Swedish Order of Freemasons Foundation Barnhuset, The Sven Jerring Foundation, The Hesselman foundation, The Magnus Bergwall foundation, The Konsul Th C Bergh’s Foundation, The Swedish Society of Medicine, The King Gustaf V 80th Birthday Foundation, KI grants, The Cancer- and Allergy Foundation, The Pediatric Research Foundation at Astrid Lindgren Children’s Hospital, The Samaritan Foundation for Pediatric research, Barnestiftelsen at Oslo University Hospital, Roche, The Frithjof Nansen Institute. The funding sources had no influence on study design, conduct, or analyses.

## Data availability

Data described in the manuscript, code book, and analytic code will be made available upon request pending application and approval.
