# Supplementary material for: Feeding Practices and Dietary Diversity in the First Year of Life: PreventADALL, a Scandinavian Randomized Controlled Trial and Birth Cohort Study
Source: J Nutr. 2023 Jun 17;153(8):2463–71. doi: 10.1016/j.tjnut.2023.06.015 (PMC10447610; doi:10.1016/j.tjnut.2023.06.015)
Supplement: Multimedia component 1 [file mmc1.docx]

1. Supplementary table, Baseline characteristics of study population and remaining PreventADALL cohort

| Characteristics parents |  | Study population  n= 2059 | Remaining cohort  n= 338 |
| --- | --- | --- | --- |
| Age mother | | 32.5 (4.1, 20-48) | 31.7 (4.6, 21-47) |
| Age father | | 34.8 (5.5, 21-72) | 33.9 (5.4, 21-55) |
| Characteristics infant |  |  |  |
| Male gender, infants |  | 1078 (52) | 183 (55) * |
| Birth weight, grams | | 3577 (476, 1794-5632) | 3564 (508, 1935-4956) |
| Birth length, cm | | 50.5 (2.1, 33-61) | 50.4 (2.2, 42-58) |
| Vaginal delivery |  | 1718 (84) | 270 (80) |
| Caesarian section |  | 328 (16) | 65 (19) |
| Other background characteristics | | | |
| Marital status^2^ | Married | 785 (41) | 110 (41) |
|  | Cohabitants | 1071 (56) | 146 (55) |
|  | Single | 34 (2) | 7 (3) |
|  | Divorced/ Separated | 0 (0) | 1 (0.5) |
|  | Other | 14 (<1) | 3 (1) |
| Maternal education level^3^ | Preliminary school (9/10y) | 11 (<1) | 5 (2) |
|  | High school only | 182 (10) | 41 (15) * |
|  | Higher education <4y | 590 (31) | 100 (38) * |
|  | Higher education >4 y | 1055 (56) | 116 (44) * |
|  | PhD | 56 (3) | 4 (2) |
|  | Other education | 2 (<1) | 0 (0) |
| Paternal education level^4^ | Preliminary school (9/10y) | 22 (1) | 4 (2) |
|  | High school only | 332 (18) | 61 (24) * |
|  | Higher education <4y | 544 (30) | 85 (33) |
|  | Higher education >4 y | 849 (46) | 98 (38) * |
|  | PhD | 63 (3) | 7 (3) |
|  | Other education | 4 (<1) | 0 (0%) |
|  | None of the above | 16 (<1) | 2 (<1%) |
| Maternal Work | Fulltime | 1629 (79) | 209 (62) * |
|  | Part-time | 168 (8) | 28 (8) |
|  | Student | 115 (6) | 23 (7) |
|  | Housewife/ homemaker | 16 (<1) | 4 (1) |
|  | Jobseeker/ unemployed | 20 (1) | 8 (2) |
|  | Disabled | 10 (0.5) | 1 (0.6) |
|  | Other | 31 (2) | 11 (3) |
| Gross income household | Below 300 000 | 17 (<1) | 8 (3) * |
| (NOK)^5^ | 300 000 -600 000 | 229 (12) | 48 (18) * |
|  | 600 000- 1 000 000 | 779 (41) | 105 (39) |
|  | 1 000 000 – 1 400 000 | 618 (33) | 70 (26) * |
|  | > 1 400 000 | 231 (12) | 29 (11) |
|  | Did not want to answer | 30 (2) | 7 (3) |
| Tobacco use | Smoking | 410 (20) | 69 (20) |
| (previous and/or current) | Snus | 423 (22) | 65 (19) |

^1^Values are means (SD, min-max) or n (%) unless otherwise stated, ^2^ Information available from n=1904/ n=267, ^3^ n=1896/ n=266, ^4^ n=1830/ n=257, ^5^ n=1904/n=267

* indicates a statistically significant difference, p>0.05

**Dietary data**

Questions regarding breastfeeding were asked retrospectively. At 3 months, parents were asked how the infant was fed the past 2 weeks with the alternatives: 1. only breastfed, 2. breastmilk, also from bottle, 3. breastfed/breastmilk and other food (including formula), 4. only other food (including formula). To indicate what types of other foods were used, follow-up questions were given with alternatives: “infant formula”, “solid foods (not from a bottle)” and “other”. If option 3 was chosen the second follow-up questions was: How much of the infant´s diet is breastmilk? The possible answers were: “most of the diet”, “same amount as other food” and “a small part of the diet”.

At 6, 9- and 12-months parents were asked about breast-feeding the past 3 months and offered the following alternatives: 1. Yes, still receiving breastmilk/ breastfed, 2. Yes, but not anymore, 3. No. Only at 6 months, if option 1 was chosen the follow-up questions was: How much of the infant´s diet is breastmilk now? The possible answers were: “no breastmilk”, “small part of the diet”, “approximately half of the diet”, “most of the diet”. At 6, 9 and 12 months of age, if option 2 was chosen parents were asked to indicate at what month the infant stopped breastfeeding/receiving breastmilk.

At 3- and 6-months parents were asked if the infant had been given porridge, how often it was given, at what age it was first introduced and what it was made of (rice, millet, oat, corn, wheat, whole meal, spelt, Sinlac, other type). Questions about dairy intake at 6, 9 and 12 months, included time of first introduction and what kind of dairy products were given to the infant. and a selection of the following options: “normal cow’s milk (full fat, reduced fat, extra light, cream etc.)”, “lactose-free milk”, “unpasteurized milk”, “buttermilk, and/or other dairy products with pre/probiotics”, “yoghurt and/or other fermented dairy products”, “cheese”, “other dairy products”.

At 6, 9- and 12-months parents were asked to indicate the consumption of solid food items, including: “bread, cookies, waffles, cakes and other bakery products”, “fruit or berries”, “root fruits (such as potato, turnip, carrot, parsnip)”, “other vegetables”, “peanuts (as spread or in other foods)”, “nuts, except for peanut (as spread or in other foods)”, “pure egg (e.g. fried, cooked, scrambled, frothy), “egg in other food (e.g. gratin, waffles, bakery, pâté)”, “ fatty fish (salmon, trout, mackerel, halibut, eel)”, “other fish”, “shellfish”, “poultry”, “other meat”. Further parents were asked to indicate how often the infant consumed these foods. the frequency at which these were given: “not at all”, “less than weekly”, “1-3 x per week”, “4 x per week”, “not at all”.
